# Emergency Absentee Voting for Hospitalized Patients and Voting During COVID-19: A 50-State Study

**DOI:** 10.5811/westjem.2021.4.50884

**Published:** 2021-07-15

**Authors:** Oliver Y. Tang, Kelly E. Wong, Reetam Ganguli, Keyana Zahiri, Nicole M. Burns, Saba Paracha, Giovanni Kozel, Kevin P. Tang, Jeremiah D. Schuur

**Affiliations:** * Warren Alpert Medical School of Brown University, Providence, Rhode Island; † Warren Alpert Medical School of Brown University, Department of Emergency Medicine, Providence, Rhode Island; ‡ Yale University, New Haven, Connecticut

## Abstract

**Introduction:**

Voters facing illness or disability are disproportionately under-represented in terms of voter turnout. Earlier research has indicated that enfranchisement of these populations may reinforce the implementation of policies improving health outcomes and equity. Due to the confluence of the coronavirus 2019 (COVID-19) pandemic and the 2020 election, we aimed to assess emergency absentee voting processes, which allow voters hospitalized after regular absentee deadlines to still obtain an absentee ballot, and election changes due to COVID-19 in all 50 states.

**Methods:**

We performed a cross-sectional study collecting 34 variables pertaining to emergency voting processes and COVID-19-related election changes, including deadlines, methods of submission for applications and ballots, and specialized services for patients. Data were obtained from, in order of priority, state boards of elections websites, poll worker manuals, application forms, and state legislation. We verified all data through direct correspondence with state boards of elections.

**Results:**

Emergency absentee voting processes are in place in 39 states, with the remaining states having universal vote-by-mail (n = 5) or extended regular absentee voting deadlines (n = 6). The emergency absentee period most commonly began within 24 hours following the normal absentee application deadline, which was often seven days before an election (n = 11). Unique aspects of emergency voting processes included patients designating an “authorized agent” to deliver their applications and ballots (n = 38), electronic ballot delivery (n = 5), and in-person teams that deliver ballots directly to patients (n = 18). Documented barriers in these processes nationwide include unavailable online information (n = 11), restrictions mandating agents to be family members (n = 7), physician affidavits or signatures (n = 9), and notary or witness signature requirements (n = 15). For the November 2020 presidential election, 12 states expanded absentee eligibility to allow COVID-19 as a reason to request an absentee ballot, and 18 states mailed absentee ballot applications or absentee ballots to all registered voters.

**Conclusion:**

While 39 states operate emergency absentee voting processes for hospitalized voters, there are considerable areas for improvement and heterogeneity in guidelines for these protocols. For future election cycles, information on emergency voting and broader election reforms due to COVID-19 may be useful for emergency providers and patients alike to improve the democratic participation of voters experiencing illness.

## INTRODUCTION

Earlier research indicates that Americans with significant health conditions or belonging to marginalized populations are disproportionately under-represented in terms of voter turnout.^1^–^6^ Healthcare institutions have the potential to improve democratic participation,^2^,^7^,^8^ and one method to achieve this is emergency absentee voting. The emergency absentee voting process allows voters to obtain and submit an absentee ballot if they experience a medical emergency or are hospitalized after their state’s regular absentee deadline, which usually falls days or weeks before election day. However, guidelines and restrictions vary greatly between states.

For elections in 2020, existing disparities in voting accessibility were challenged further by the ongoing coronavirus disease 2019 (COVID-19) pandemic. Infections caused by the severe acute respiratory syndrome coronavirus 2 have been diagnosed in over 28 million cases in the United States (US), with over 500,000 deaths thus far.^9^ Moreover, leading up to the election, an estimated 5,000–10,000 new hospitalizations daily occurred due to COVID-19.^10^ Significant disparities in disease impact and mortality have been documented not only in older populations and those with comorbidities, but also across racial and socioeconomic lines.^11^,^12^ This rise in hospitalizations may have increased the utilization and value of emergency absentee processes for patients unable to attend the polls in-person. The current pandemic also created challenges for all voters in general.

Among several studies documenting “superspreading” events due to large public gatherings,^13^–^15^ some studies have suggested that elections may also be linked to increased viral transmission^16^,^17^; however, evidence on these surges has been mixed.^18^ Nevertheless, in 2020 state governments implemented election delays and varying changes to voting processes for statewide and national elections, such as mailing ballots or ballot applications to voters and temporarily switching to universal mail-in voting. The confluence of the November 2020 election and COVID-19 emphasized the importance for patient and provider awareness of remote voting mechanisms that may both ensure access to voting for hospitalized voters and ameliorate the viral transmission risks by providing an alternative to in-person voting. However, to the best of our knowledge, there has been no nationwide assessment of emergency absentee voting processes or election changes nationwide due to COVID-19. Consequently, in the present study we aimed to a) profile state-by-state details and national trends in “emergency absentee processes” available to hospitalized voters and b) summarize changes in all 50 states’ overall voting processes in light of COVID-19.

## METHODS

We collected 34 variables related to emergency absentee voting processes and election changes implemented due to COVID-19 for all 50 states from July15–November 3, 2020. Collected variables were determined using both deductive and inductive approaches.^19^ Two authors (OYT and KEW) collated an initial set of variables from a first review of elections websites for all 50 states. This variable list was iteratively expanded through the the process of the study’s data collectors convening weekly during data collection to discuss emergent themes across statewide protocols, representing new variables to record, until thematic saturation was reached. In order of priority we obtained data for these variables from each state’s board of elections website, poll worker manuals, application forms, and state legislation. Variables related to election process changes implemented due to COVID-19 were re-collected weekly, due to the evolving nature of these changes. We verified collected data through correspondences with the boards of elections of all 50 states. Washington, DC, was not included for analysis due to nonresponse from the District of Columbia Board of Elections. This study was exempt from institutional review board approval, due to the publicly available nature of these data.

**Population Health Research Capsule**

What do we already know about this issue?

*United States’ citizens with health conditions have significantly lower voter turnout. In several states, emergency absentee voting enables hospitalized patients to vote*.

What was the research question?

*What statewide processes are available for unexpectedly hospitalized patients to access an absentee ballot?*

What was the major finding of the study?

*A total of 39 states have emergency absentee voting processes, with varying deadlines, features, and barriers to access*.

How does this improve population health?

*Emergency absentee voting may improve democratic participation among voters facing significant health conditions and promote more equitable policymaking*.

We report details for each state’s emergency absentee voting process representing important information for physicians and patients to be aware of, including deadlines, methods of submission for applications and ballots, and specialized services such as in-person, ballot delivery teams. We used descriptive statistics and color-coded maps to summarize shared characteristics across states. All analyses were performed using Stata 15 (StataCorp, College Station, TX).

## RESULTS

### National Overview of Absentee and Emergency Absentee Voting

**Figure 1:**
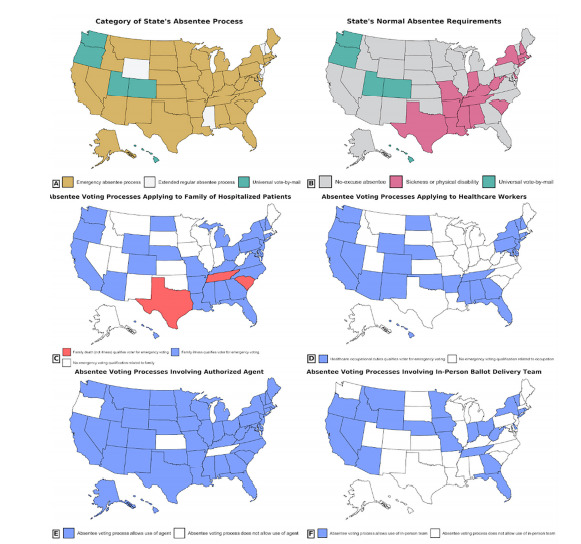
Nationwide map of state absentee voting practices. A. Nationwide distribution of absentee voting categories (universal vote-by-mail, no-excuse absentee voting, or absentee voting requiring an excuse). B. States with emergency absentee voting processes. C. States with absentee voting processes also applying to family members of hospitalized patients. D. States with absentee voting processes also applying to healthcare workers. E. States incorporating the use of an authorized agent for the voter. F. States using in-person ballot delivery teams.

Twenty-nine states have no-excuse absentee voting systems, wherein no excuse or condition is required to obtain an absentee ballot (Figure 1A). Of the remaining 21 states, five conduct universal vote-by-mail elections, whereas 16 require specific conditions, such as physical disability or hospitalization to apply for an absentee ballot. However, because the deadline to apply for an absentee ballot is often days or weeks before election day, 39 states have “emergency” absentee voting processes for voters experiencing a medical emergency or hospitalization after this deadline (Figure 1B). The remaining six non-universal, vote-by-mail states did not have legislation on emergency absentee voting but were classified as having “extended regular absentee processes,” due to having deadlines falling within 24 hours of election day or not having any specific application deadline. While emergency absentee processes primarily serve hospitalized voters, 23 states also had legislation extending emergency absentee voting privileges to family members of hospitalized patients, and 17 states had such legislation for healthcare workers unable to vote due to occupational duties (Figures 1C–D).

### Steps of Emergency Absentee Voting Process and Interstate Differences

The normal absentee application deadline was most commonly seven days before an election (11 states), but this deadline ranged from 21 days (Rhode Island) to one day (four states) before an election. (Supplementary Figures A–B and Supplementary Table 1). For the 39 emergency absentee voting processes nationwide, the emergency absentee period most commonly began within 24 hours following the normal absentee application deadline. Only 28 states had publicly available information on their board of elections website outlining the state’s specific protocol.

**Figure 2:**
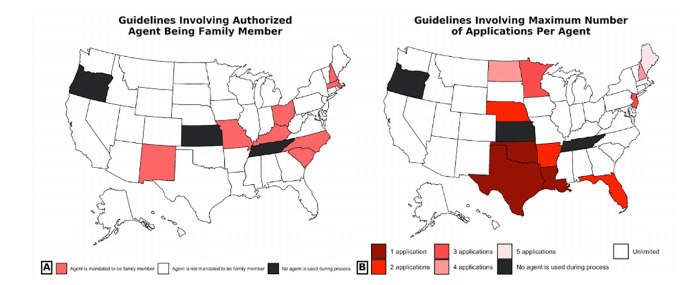
Statewide rules for voter’s authorized agent. A. Rules concerning whether a voter’s authorized agent is mandated to be a family member. B. Rules concerning the maximum number of applications or ballots that a single agent can handle during an election. ^a^This regulation may vary county by county within the state. ^b^In Florida, the maximum limit of two applications per agent does not include immediate family members of the agent.

The procedure for obtaining and voting through an emergency absentee ballot entails three steps. First, a hospitalized voter must fill out an initial emergency absentee application. Twenty-five states allow applications to be directly downloaded from the board of elections website, but the remaining states necessitate contacting a local election official to obtain an application. Moreover, nine states mandate a physician signature or affidavit on the application, attesting to the voter’s hospitalization (Supplementary Table 2). The voter must subsequently return their filled-out application to their local election official (Table). The most common submission method is through an authorized agent (38 states), wherein the voter appoints an “agent,” a representative designated for delivering the application in person (Figure 1E). Seven states mandate that a voter’s agent must be a family member, but anyone, such as a healthcare worker, may serve as an agent in the remaining 31 (Figure. 2A). Additionally, 29 states do not limit the maximum number of applications a single agent can process (Figure 2B). Twenty-five states alternatively allow for applications to be submitted by mail, and 21 states have electronic submission methods such as email, fax, or phone requests. Applications must be returned by a specific deadline, which may fall 24–48 hours earlier than the eventual ballot return deadline (Supplementary Figure A).

Second, the voter must obtain their emergency absentee ballot. Thirty states allow for the voter’s agent to pick up and return the ballot, following the processing of the emergency application. Alternatively, 17 states can mail the ballot to a voter’s hospital, and five states can electronically deliver a ballot such as through an online voter portal. Finally, 18 states may send bipartisan, in-person teams to deliver ballots directly to hospitalized voters (Figure 1F). These teams automatically return a voter’s ballot to be counted after it has been filled out. However, in 10 of these states, the accessibility of in-person teams varies depending on where the voter is hospitalized (Supplementary Table 3).

Third, and finally, the voter must fill out and return their ballot. Fifteen states normally require a notary or witness to sign the absentee ballot before it can be counted, with a notary being the only option in four states (Supplementary Table 4). Voters may return their ballot through their agent (32 states), the mail (23 states), or an in-person ballot delivery team (18 states). Across all 39 states, the ballot return deadline falls after 12 pm on election day (Supplementary Figure A).

### Accommodations for Hospitalized Voters in States Without Emergency Processes

The six states with extended regular absentee processes have absentee applications deadlines within 24 hours of election day (Supplementary Figure B). Despite not having formal emergency absentee processes these states often had components of these procedures, such as allowing voters to use authorized agents and using electronic and in-person team delivery of ballots (Supplementary Table 5). Additionally, the five states with universal vote-by-mail that mail ballots to all registered voters have processes for voters to re-obtain a ballot if they are separated from their original ballot due to a situation such as hospitalization.

### Election Changes Made Due to COVID-19

**Figure 3:**
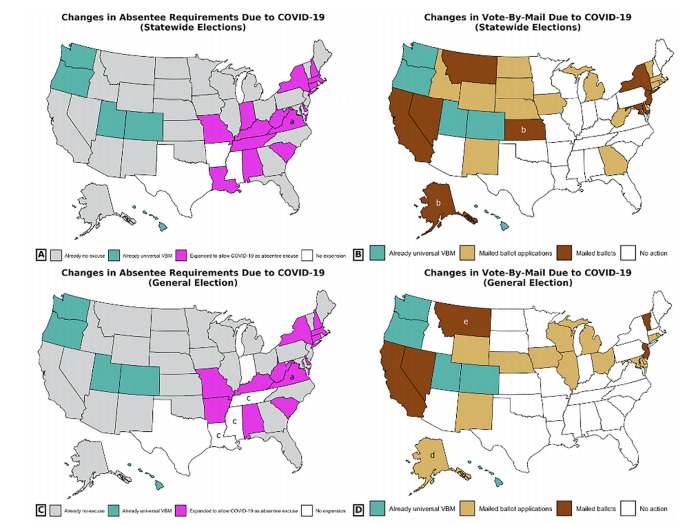
Nationwide map of election process changes due to COVID-19. A. Expansion of absentee voting eligibility during state-level elections before November 2020. B. Expansion of mail-in ballots and application during state-level elections before November. C: Expansion of absentee voting eligibility for the November general election. D: Expansion of mail-in ballots and applications for the November general election. a Virginia was already deliberating legislation to make absentee voting no-excuse before the COVID-19 pandemic, with an anticipated start date of July 1, 2020, but the state implemented this change earlier for its May municipal elections. b These changes only applied to a presidential primary for a specific party and were not made by the state government. c While absentee voting was not expanded to no-excuse in Louisiana, Tennessee, and Mississippi, these three states introduced absentee eligibility for voters under quarantine, serving as caretakers for others under quarantine, or belonging to a high-risk group for COVID-19. d Absentee ballot applications were only mailed to voters above the age of 65. e Montana allowed individual counties to make the choice to mail voters absentee ballots. *VBM*, vote-by-mail.

In response to COVID-19, 20 states delayed state-level elections in 2020, such as congressional primaries. In the 16 states requiring specific conditions to apply for an absentee ballot, 13 states expanded absentee eligibility to allow social distancing or concerns over COVID-19 as a legitimate excuse to obtain an absentee ballot (Figure 3A). Among the 45 states without universal vote-by-mail, 15 mailed absentee ballot applications and eight mailed absentee ballots to all registered voters (Figure 3B). Finally, of the 15 states with notary- or witness-signature requirements, eight loosened these regulations due to COVID-19, while seven did not make any changes (Supplementary Table 4).

Only a fraction of these changes applied to the November 2020 general election. Only 12 states continued to expand absentee eligibility requirements due to COVID-19 (Figure 3C). Thirteen states and five states mailed absentee ballot applications or absentee ballots, respectively, for the general election (Figure 3D). Six states extended the receipt deadline for receiving mail-in ballot deadlines (Supplementary Table 2), but similar efforts in Michigan and Wisconsin were overturned by federal courts.

Finally, COVID-19 also impacted emergency absentee processes within certain states. For example, the state of Maryland temporarily canceled in-person ballot requests, due to local election offices being closed to the public. Additionally, in light of infection control-related restrictions to hospital visitor regulations, election officials in six states reported the cancellation or decreased use of in-person ballot delivery teams (Arizona, Iowa, New York, Rhode Island) or in-hospital election workers to assist patients with ballots (Alaska and Minnesota) for 2020 state-level elections. However, four states (Arizona, Rhode Island, Tennessee, and Texas) reported adapting to these restrictions by swearing in or involving healthcare workers in ballot delivery teams.

## DISCUSSION

In the setting of evidence that voters facing illness or disability are under-represented at the ballot box,^1^–^6^ a potential way to improve democratic participation among this population is emergency absentee voting. These protocols allow hospitalized or ailing individuals to obtain ballots after the regular absentee deadline. Over three-quarters of states have an emergency absentee process, while the remaining have comparatively later regular absentee ballot deadlines or, in the case of universal mail-in ballot states, have last-minute replacement ballot options. The current study’s summary of emergency absentee ballot procedures demonstrated a canonical process across states: patients must first submit an application; secondly, obtain their ballot; and, finally, return their filled-out ballot.

We found considerable heterogeneity between states in the sum of options, instructional clarity, and level of nuance for emergency absentee voting. A notable accommodation within emergency absentee processes is the use of a designated agent to carry out each step of the process. In particular, the majority of states do not require a voter’s agent to be a family member or limit the maximum number of applications an agent can handle, allowing healthcare or social workers to potentially facilitate this process for patients. Moreover, 18 states employ in-person teams to deliver ballots directly to patients and eventually return them. Electronic means for application submission and ballot delivery may also expedite emergency voting processes, but the extremely limited use of these methods indicates substantial room for expansion. Nevertheless, the present analysis also highlights notable areas of improvement for emergency voting processes, with the first being lack of access to public information.

Eleven states with emergency processes do not have this information on their board of elections websites, and 14 states do not have emergency ballot applications readily available for download. These processes also have substantial geographic variability. For example, over half of states with an in-person team delivery option have geographic restrictions determining whether a team can be sent to a voter. Requirements of designating family members as agents (seven states) also impede emergency voting for patients without readily available family. Administrative obstacles exist as well; several states have a notary and/or witness requirement for emergency absentee voting, and many also require a physician affidavit. In the most onerous case, Arkansas does not accept physician validation and requires a signature from a hospital’s administrative head. Conversely, in North Carolina it is a felony for hospital employees to assist patients with absentee voting.^20^

Limited studies have analyzed the issue of voting while hospitalized, and earlier research has primarily focused on assessing competency for certain hospitalized populations, such as patients with dementia, and the under-representation of patients in the voting population.^8^,^21^,^22^ A common finding from the literature is that ill patients may have different voting priorities than the general population, especially on matters related to healthcare.^4^,^23^ An under-representation of these voters may impact policy decisions pertaining to medical care and population health, and some studies have accordingly called for healthcare workers to address barriers to voting faced by patients.^2^,^7^,^8^,^24^–^27^ Importantly, several studies have indicated that enfranchising marginalized populations is associated with improved health outcomes, due to these voters disproportionately supporting policies focused on equity, including healthcare and education.^1^,^23^,^28^

There are several ways that healthcare workers and institutions may act on this study’s findings. First, healthcare workers may strive to educate themselves on the specific absentee and hospitalized voting procedures for their state — such as absentee ballot requirements and deadlines as well as methods of ballot and application delivery, especially given substantial interstate heterogeneity — and counsel interested patients accordingly, particularly those expressing concerns about missing an election due to their hospitalization. Healthcare workers should navigate the topic of voting with their patients akin to obtaining informed consent for a procedure, and they should respect a patient’s decision to abstain from voting. Hospitals may also seek to expand patient knowledge by distributing informational flyers and codifying discussions of emergency voting into care encounters, such as social work consultations.

Second, hospital personnel may aim to improve the convenience of the documentation necessary for emergency voting, through measures such as printing out readily available ballot applications, coordinating mailing services, and arranging notary services for states with these requirements. Third, in states where it is allowed, healthcare workers may serve as agents for voters without any available designee, such as by delivering a patient’s absentee application or assisting a ballot delivery team looking for the patient. Hospitals may also target volunteer recruitment toward this specific purpose. Fourth, hospitals may seek to partner directly with their local election body to establish a formalized process for patients to undertake absentee voting, a communication line for any troubleshooting or process updates, and institutional experience across election cycles. These recommendations may be especially important for emergency physicians, who are most commonly the first-line providers for unexpectedly hospitalized patients. Increasing public awareness of and access to emergency voting processes may improve representation of hospitalized voters.

Additionally, COVID-19 drove several states to make notable changes to overall election processes during 2020, including switching to entirely universal vote-by-mail elections, mailing absentee ballot applications or ballots to all registered voters, expanding absentee ballot eligibility to include concerns over COVID-19, and reducing notary/witness requirements; however, the carryover of these changes from statewide elections to the November 2020 general election was more limited. The pandemic also limited the operation of emergency absentee voting in several states. For example, to mitigate risk six states canceled the use of in-person teams but two (Arizona and Rhode Island) reported implementing teams using sworn-in healthcare workers to deliver ballots. It is conceivable that increases in hospitalizations due to COVID-19 increased the utilization of emergency absentee voting processes, but limited data in emergency ballot counts for most states limited analysis of this. However, in our anecdotal experience coordinating a nonpartisan emergency absentee voting organization called Patient Voting,^29^ over 50% of patient inquiries nationwide were related to COVID-19 hospitalizations.

The COVID-19 pandemic presented several public health implications for the November 2020 election. Among several studies elucidating a potential link between elections and rises in viral transmission,^16^,^17^ long-distance absentee voting options, which have been empirically demonstrated to have no impact on partisan turnout and minimal risk for fraud,^30^,^31^ were increasingly used. An estimated 65 million mail-in ballots were cast in the 2020 election, compared to 33.5 million in 2016, which may have contributed to the historic turnout rate of over 65%.^32^,^33^ The evolving election changes and the variable availability of voting-related information documented in this study emphasize the importance of state boards of elections clearly communicating voting processes to the public well in advance of elections.

Nevertheless, states faced additional infrastructure challenges for sufficiently handling an influx of mail-in ballots, which may continue to hold importance for future elections. Online portals for voters to request and track absentee ballots warrant expansion, such as incorporating notification of potential marking issues. Additionally, given research demonstrating that limited in-person voting options may disproportionately disenfranchise marginalized populations,^34^ states still need to maintain in-person elections in some capacity for populations such as voters without internet access or requiring assistance due to disability. For 2020, this required investment into increased poll worker hiring, personal protective equipment for voters and workers, and sanitization resources for voting facilities and machines. While quantifying the nationwide costs of these 2020 election resilience measures is the subject of future study, one report projected that these reforms may have cost approximately $2 billion dollars.^35^

## LIMITATIONS

The present study has several potential limitations. First, nearly every state did not track the number of emergency absentee ballots cast in elections, as these counts were often aggregated with general absentee voting turnout. Consequently, we were unable to assess variables associated with statewide differences in emergency absentee voting turnout or longitudinal trends in emergency absentee voting. Nevertheless, certain states such as Pennsylvania anecdotally reported increases in emergency absentee turnout following legislation simplifying requirements such as application submission methods. To facilitate future research on emergency absentee processes, such as characteristics that may influence turnout, states should record this outcome.

Second, the constant evolution of voting procedures due to COVID-19 complicated the process of collecting these variables, as states’ disparate legal landscapes and state government decision-making produced varying levels of expansion, including some measures being reversed. Nevertheless, the data presented represents the information available at the time of the 2020 general election being conducted and may serve to guide public health dialogue on these measures. Third, because state emergency voting policies are actively evolving, some of the information in this study may not apply to future election cycles. Due to COVID-19 impacting even permanent election policy for some states, such as motivating Virginia’s decision to expedite its transition to no-excuse absentee voting, it is conceivable that some changes made in 2020 due to the pandemic may be permanently extended into future elections by legislation. We believe our findings are significant in that they a) explain the archetypal process of emergency absentee voting for patients; b) summarize the current state of emergency absentee voting nationwide; and c) elucidate barriers to voting that future legislation may aim to alleviate.

## CONCLUSION

This study reports information on emergency absentee voting for physicians and patients and summarizes information on 2020 election changes driven by the COVID-19 pandemic. We report nationwide data on election processes for physicians to mitigate the impact on marginalized and under-represented populations disproportionately affected by healthcare disparities. The COVID-19 pandemic has proven the necessity of voting systems structured to assist patients burdened by illness and disability. Understanding emergency voting procedures for sick or hospitalized voters is an important step.

## Supplementary Information





## Figures and Tables

**Table:** Methods for submitting application, obtaining ballot, and returning ballot.

State	Methods to submit application			Methods to obtain ballot				Methods to submit ballot	
		
	Agent	Mail	Electr.	Agent	Mail	Electr.	IPT	Agent	Mail
Alabama	X	X		X				X	
Alaska	X			X				X	
Arizona	X	X	X				X		
Arkansas	X	X	X	X	X			X	X
California	X			X			X^d^	X	
Connecticut	X	X		X	X		X	X	X
DC	X			X				X	
Florida	X		X^a^	X			X^d^	X	X
Georgia	X^a^	X	X	X^a^	X	X	X^d^	X^a^	X
Idaho	X	X	X		X		X^d^		X
Illinois	X			X				X	
Indiana	X	X	X				X		
Iowa	X^b^	X^b^	X^b^		X^b^		X^b^,^d^	X	X
Kansas		X	X		X				X
Kentucky	X	X	X	X	X			X	X
Louisiana	X	X	X	X	X	X		X	X
Maine	X			X				X	X
Maryland	X			X		X^c^		X	X
Massachusetts	X	X	X	X	X			X	X
Michigan	X			X				X	
Minnesota	X	X	X	X	X		X^d^	X	X
Missouri	X	X	X	X	X		X^d^	X	X
Montana	X		X	X			X	X	
Nebraska	X	X	X	X	X	X	X	X	X
Nevada	X	X	X	X			X	X	X
New Mexico	X	X	X		X	X		X	X
New York	X			X			X^d^	X	
North Carolina	X			X				X	X
North Dakota	X	X	X	X	X			X	X
Ohio	X	X		X	X		X^d^	X	
Oklahoma	X	X	X	X	X			X	X
Pennsylvania	X	X		X	X			X	X
Rhode Island	X	X					X		
South Carolina	X			X				X	
South Dakota	X	X		X				X	X
Tennessee	X	X	X				X		
Texas	X			X				X	
Virginia	X			X				X	
West Virginia	X	X	X				X		
Wisconsin	X			X				X	

Breakdown of possible methods for submitting the emergency absentee application, obtaining the ballot, and returning the filled-out ballot for all 40 emergency absentee voting processes nationwide. “X” denotes that this is a viable method within the state.

a This method may not be universally available across all counties within the state and the patient should clarify with their county election office whether this method is allowed.

b Iowa’s emergency absentee voting process has several phases. A voter hospitalized before 10/24 5 PM can submit an application by mail or agent to obtain an absentee ballot by mail. A voter hospitalized after this time but before 10/30 5 PM may follow the same submission methods to obtain an absentee ballot through an in-person team. Finally, voter hospitalized on 10/31 or after may contact their county auditor directly, such as by phone or email, to obtain an absentee ballot through an in-person team.

c Electronic delivery of emergency absentee ballots in Maryland is possible but decided on a case-by-case basis.

d In-person ballot delivery teams are only available based on certain geographic or institutional requirements, which are detailed in Supplementary Table 3.

*DC*, District of Columbia; *Electr.*, electronic; *IPT*, in-person team.
