# Supplementary material for: Emergency Absentee Voting for Hospitalized Patients and Voting During COVID-19: A 50-State Study
Source: West J Emerg Med. 2021 Jul 15;22(4):1000–9. doi: 10.5811/westjem.2021.4.50884 (PMC8328173; doi:10.5811/westjem.2021.4.50884)
Supplement: Supplementary file 1 [file wjem-22-1000-s001.docx]

**
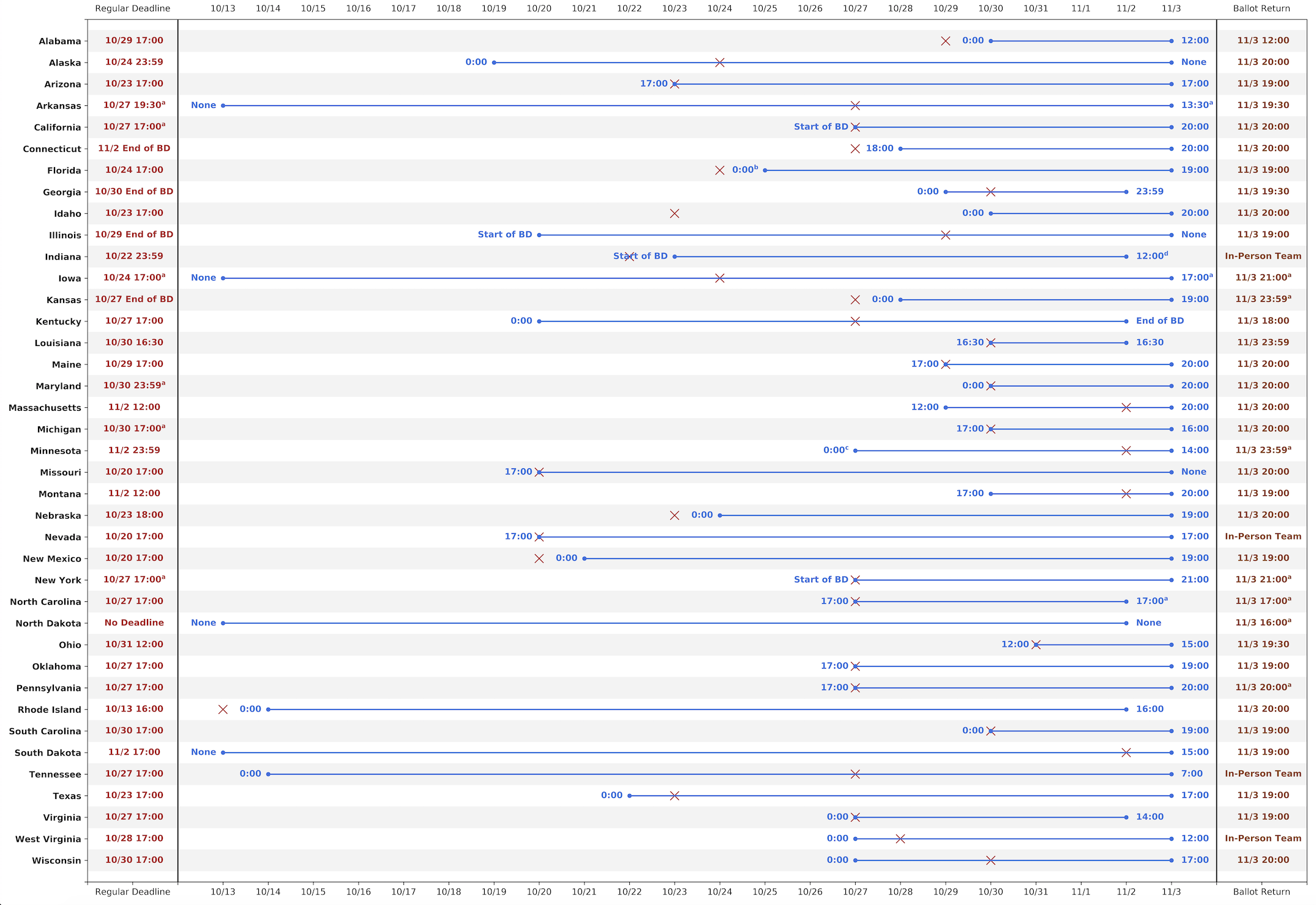
**

**
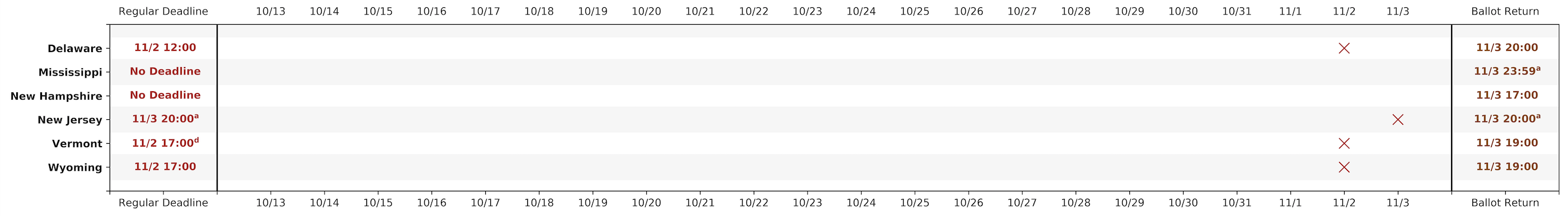
**

**Supplementary Figure.** Important Deadlines for Emergency Absentee Voting Processes

**A:** Deadlines for emergency absentee voting in 39 states and DC. The left column and red X indicate the deadline for normal absentee applications. The blue line and times indicate the beginning (left) and end (right) of the emergency voting period. The right column indicates the deadline for ballot return. In 4 states, the ballot delivery team is the only possible method of returning the emergency ballot. All times are displayed in the 24-hour convention and for the state’s local time. **B:** Deadlines for absentee voting in the 6 states with extended regular absentee processes. The left column and red X indicate the deadline for absentee applications. The right column indicates the deadline for ballot return.

BD = business day

^a^ For this state, multiple deadlines exist depending on the method of submission or there may be special rules about mail postmarking and arrival deadlines. For this figure, the earliest deadline that did not require the voter to be present in-person was reported. All possible deadlines were recorded in Supplementary Table 1.

^b^ In Florida, the start time of the emergency absentee voting period may be later than 10/25 0:00, depending on the county.

^c^ In Minnesota, an in-person ballot delivery team may alternatively be requested by a hospitalized voter as early as 10/14 0:00.

^d^ In this state, a voter’s application for an absentee ballot may potentially be accepted after the indicated deadline on a case-by-case basis.
