# Supplementary material for: Emergency Absentee Voting for Hospitalized Patients and Voting During COVID-19: A 50-State Study
Source: West J Emerg Med. 2021 Jul 15;22(4):1000–9. doi: 10.5811/westjem.2021.4.50884 (PMC8328173; doi:10.5811/westjem.2021.4.50884)
Supplement: Supplementary file 2 [file wjem-22-1000-s002.docx]

**Supplementary Table 1: Alternative Deadlines for Absentee Voting**

| **State** | ***Normal* Absentee Application Deadlines** | ***Emergency* Absentee Application Deadlines** | **Absentee Ballot Return Deadlines** |
| --- | --- | --- | --- |
| Arkansas | 10/27 19:30 (mail or electronic)  11/2 end of BD (in-person) | 10/27 19:30 (mail or electronic)  11/3 13:30 (agent) |  |
| California | 10/27 17:00 (mail)  11/3 20:00 (in-person) |  |  |
| Iowa | 10/24 17:00 (mail)  11/2 17:00 (in-person) | 10/30 17:00 (agent)  11/3 17:00 (electronic) | 11/3 21:00 (agent)  11/3 21:00 (mail): If received after this time, the ballot must be postmarked by 11/2 and arrive by 11/9 12:00 |
| Kansas |  |  | 11/3 23:59 (mail): The ballot must be postmarked by 11/3 and arrive by 11/6 |
| Maryland | 10/27 end of BD (mail or electronic): Deadline to receive ballot by mail or fax  10/30 23:59 (electronic): Deadline to receive ballot through online portal |  | 11/3 20:00 (agent)  11/3 23:59 (mail): The ballot must be postmarked by 11/3 and received by 11/13 10:00 |
| Michigan | 10/30 17:00 (mail)  11/2 16:00 (in-person) |  |  |
| Minnesota |  |  | 11/3 23:59 (mail): The ballot must be postmarked by 11/3 and arrive by 11/10 end of BD^a^ |
| Mississippi |  |  | 10/31 12:00 (agent)  11/3 23:59 (mail): The ballot must be postmarked by 11/3 and arrive by 11/8 end of BD^a^ |
| New Jersey | 10/27 23:59 (mail)  11/3 20:00 (in-person) |  | 11/3 20:00 (agent)  11/3 23:59 (mail): The ballot must be postmarked by 11/3 and arrive by 11/9 end of BD^a^ |
| New York | 10/27 17:00 (mail)  11/2 17:00 (in-person) |  | 11/3 21:00 (agent)  11/3 23:59 (mail): The ballot must be postmarked by 11/3 and arrive by 11/10 end of BD^a^ |
| North Carolina |  |  | 11/3 17:00 (agent)  11/3 23:59 (mail): The ballot must be postmarked by 11/3 and arrive by 11/12 17:00^a^ |
| North Dakota |  |  | 11/3 16:00 (agent)  11/2 23:59 (mail): The ballot must be postmarked by 11/2 |
| Oregon |  | 10/27 end of BD (mail or electronic)  11/3 20:00 (agent) |  |
| Pennsylvania |  |  | 11/3 20:00 (agent)  11/3 23:59 (mail): The ballot must be postmarked by 11/3 and arrive by 11/6 17:00^a^ |

Summary of all possible absentee application and ballot return deadlines for states with deadlines that differ depending on the method of submission. All times are displayed in the 24-hour convention and for the state’s local time.

BD = business day

^a^ This was an extension in the ballot receipt deadline made specifically due to COVID-19 and may not apply to future election cycles. Extensions in ballot receipt deadlines in Michigan and Wisconsin were overturned by federal courts.**Supplementary Table 2: Physician Involvement in Emergency Absentee Voting Applications**

| **State** | **Physician Signature or Affidavit Requirement** |
| --- | --- |
| Alabama | Physician affidavit |
| Arkansas | Affidavit from the administrative head of patient’s hospital or nursing home |
| Illinois | Physician affidavit |
| Louisiana | Physician affidavit |
| New Mexico | Physician signature |
| Oklahoma | Physician affidavit (requirements vary depending on county) |
| Texas | Physician affidavit^a^ |
| Virginia | Physician signature |
| West Virginia | Physician signature |

Description of requirements for the 8 states where a physician signature or affidavit is required for the voter’s emergency absentee application to be approved. An affidavit is defined as a formal statement attesting to a voter’s hospitalization or illness that has to be written by a physician.

^a^ The Texas 3rd Court of Appeals overturned a state district court order that would have temporarily suspended the physician affidavit requirement due to COVID-19.

**Supplementary Table 3: Regulations for Provision of In-Person Ballot Delivery Teams**

| **State** | **In-Person Ballot Delivery Team Regulations** |
| --- | --- |
| California | In-person ballot delivery teams are only offered by certain counties |
| Florida | In-person ballot delivery teams are only offered by certain counties |
| Georgia | In-person ballot delivery teams are only offered by certain counties |
| Idaho | In-person ballot delivery teams are only offered by certain counties |
| Iowa | In-person ballot delivery teams are only offered if the voter is hospitalized on or after 10/31 and the voter must be hospitalized in their same county of residence |
| Minnesota | In-person ballot delivery teams are only offered if the voter is hospitalized in their city or town of residence |
| Missouri | In-person ballot delivery teams are only offered if the voter is hospitalized after 10/21 17:00 and the voter must be hospitalized in their same county of residence or an adjacent county |
| New York | In-person ballot delivery teams are only offered in specific nursing home or Veteran Health Affairs institutions where more than 25 voters request a ballot to be delivered |
| Ohio | In-person ballot delivery teams are only offered if the voter is hospitalized in their same county of residence |
| Wyoming | In-person ballot delivery teams are only offered by certain counties |

Description of guidelines regulating where in-person ballot delivery teams are allowed to operate for the 10 states that have such restrictions. For example, certain states operate in-person teams on a county-by-county basis so not all counties may offer in-person teams. For the remaining states with in-person ballot delivery teams, there are no restrictions on whether the voter can request an in-person team.

**Supplementary Table 4: Notary or Witness Requirements for Absentee Voting**

| **State** | **Notary or Witness Requirements** | **Changes in Requirements Due to COVID-19** |
| --- | --- | --- |
| Alabama | Signature from notary or 2 witnesses | No change (the Supreme Court blocked a lower court’s order to loosen these requirements in 3 counties) |
| Alaska | Signature from 1 witness | No change |
| Kentucky | Signature from notary^a^ | The notary signature requirement was suspended for the June primary |
| Louisiana | Signature from 1 witness | No change |
| Maine | Signature from notary or 2 witnesses (this requirement only applies if the voter’s agent is not an immediate family member) | No change (while an executive order in April loosened certain notary or witness, this did not apply to absentee ballots ) |
| Minnesota | Signature from notary or 1 witness | The signature requirement was suspended for the August primary and general election |
| Mississippi | Signature from notary or 1 witness (witness signature is only allowed if the voter is temporarily or permanently disabled) | No change |
| Missouri | Signature from notary (this requirement does not apply if the voter is confined due to illness) | The notary requirement was suspended for voters belonging to high-risk categories, such as being over the age of 50 or having certain chronic conditions (ex. diabetes) |
| North Carolina | Signature from notary or 2 witnesses | The signature requirement was reduced from 2 witnesses to 1 for all 2020 elections |
| Oklahoma | Signature from notary | Voters are allowed to submit a copy of photo ID or their voter identification card in lieu of the signature requirement |
| Rhode Island | Signature from notary or 2 witnesses | The signature requirement was suspended for all 2020 elections |
| South Carolina | Signature from notary or 1 witness | The signature requirement was suspended for the June primary, but not the general election |
| South Dakota | Signature from notary (voters may opt out of this requirement by sending a copy of a photo ID) | No change |
| Virginia | Signature from notary or 1 witness | The signature requirement was suspended for the June primary and general election |
| Wisconsin | Signature from 1 witness | No change |

Description of notary and/or witness signature requirements for absentee ballots in the 15 states with such policies as well as changes in these requirements due to COVID-19.

^a^ The notary signature is required for the emergency absentee ballot application, not the ballot itself.

**Supplementary Table 5: Accommodations for Hospitalized Voters in Other States**

| **State** | **Accommodations for Hospitalized Voters** |
| --- | --- |
| *Colorado* | An application for a replacement ballot can be submitted by an agent or electronically, with a deadline of 11/3 17:00  The replacement ballot can be obtained by agent or electronically  The ballot can be returned by agent or electronically, with a deadline of 11/3 19:00 |
| Delaware | An application for an absentee ballot can be submitted by agent, mail, or electronically, with a deadline of 11/2 12:00  The absentee ballot can be obtained by mail, electronically, or through an in-person ballot delivery team  The ballot can be returned by agent or mail, with a deadline of 11/3 20:00 |
| *Hawaii* | An application for a replacement ballot can be submitted by mail or electronically, with a deadline of 10/27 16:30 (but this deadline can be appealed on a case-by-case basis)  The replacement ballot can be obtained by mail  The ballot can be returned by agent or mail, with a deadline of 11/3 19:00 |
| Mississippi | An application for an absentee ballot can be submitted by agent or mail, with no specific deadline  The absentee ballot can be obtained by mail  The absentee ballot can be returned by mail, with a deadline of 11/2 17:00 |
| New Hampshire | An application for an absentee ballot can be submitted by agent, mail, or electronically, with no specific deadline  The absentee ballot can be obtained by agent or mail  The ballot can be returned by agent or mail, with a deadline of 11/3 17:00 |
| New Jersey | An application for an absentee ballot can be submitted by agent (deadline of 11/3 20:00) or mail (deadline of 10/27 23:59)  The absentee ballot can be obtained by agent (anytime) or mail (if the application was submitted on or before 10/27)  The ballot can be returned by agent (deadline of 11/3 20:00) or mail (deadline detailed in Supplementary Table 1) |
| *Oregon* | An application for an absentee ballot can be submitted by agent (deadline of 11/3 20:00) or mail or electronically (deadline of 10/27 end of business day)  The replacement ballot can be obtained by agent (anytime) or mail (if the application was submitted on or before 10/27)  The ballot can be returned by agent or mail, with a deadline of 11/3 20:00  Certain institutions may form a bipartisan Facility Assistance Team to assist voters with disabilities with voting |
| *Utah* | An application for a replacement ballot can be submitted by an agent, mail, or electronically, with a deadline of 11/3 20:00  The replacement ballot can be obtained by an agent, mail, or electronically  The ballot can be returned by agent or mail, with a deadline of 11/3 20:00 |
| Vermont | An application for an absentee ballot can be submitted by agent, mail, or electronically, with a deadline of 11/2 17:00 (but this deadline can be appealed on a case-by-case basis)  The absentee ballot can be obtained by mail, electronically, or through an in-person ballot delivery team  The ballot can be returned by agent or mail, with a deadline of 11/3 19:00 |
| *Washington* | An application for a replacement ballot can be submitted by an agent, mail, or electronically, with a deadline of 11/3 20:00  The replacement ballot can be obtained by an agent, mail, or electronically  The ballot can be returned by agent or mail, with a deadline of 11/3 20:00 |
| Wyoming | An application for an absentee ballot can be submitted by agent, mail, or electronically, with a deadline of 11/2 17:00  The absentee ballot can be obtained by agent or mail  The ballot can be returned by mail, with a deadline of 11/3 19:00 |

Description of absentee voting accommodations available for hospitalized voters within the 6 states with extended regular absentee processes (non-italicized) and 5 universal vote-by-mail states (italicized). All times are displayed in the 24-hour convention and for the state’s local time.
